# Miliary-Pattern Pulmonary Tuberculosis During Infliximab Therapy Despite Negative Tuberculosis Screening

**DOI:** 10.7759/cureus.117581

**Published:** 2026-10-07

**Authors:** Adel Kenawi, Nomaan Zahid, Mohammad Humeda, Ahmed Arbab, Marwa Ruziek

**Affiliations:** 1 Cardiology, Tameside and Glossop Integrated Care NHS Foundation Trust, Ashton-Under-Lyne, GBR; 2 Internal Medicine, Tameside and Glossop Integrated Care NHS Foundation Trust, Ashton-Under-Lyne, GBR; 3 Acute Medicine, Tameside and Glossop Integrated Care NHS Foundation Trust, Ashton-Under-Lyne, GBR; 4 Acute medicine, Tameside and Glossop Integrated Care NHS Foundation Trust, Ashton-Under-Lyne, GBR

**Keywords:** active pulmonary tuberculosis, crohn’s disease, differential diagnosis, disseminated tuberculosis, extrapulmonary tuberculosis, infliximab, intestinal tuberculosis, miliary tuberulosis, mycobacterium tuberculosis infection, ulcerative colitis

## Abstract

Tumour necrosis factor-alpha (TNF-α) inhibition increases the risk of tuberculosis (TB), including disseminated and miliary disease. Although screening for latent TB is recommended before anti-TNF therapy, negative or indeterminate interferon-gamma release assay (IGRA) results do not exclude active TB, particularly in immunosuppressed patients. We report a 32-year-old man with ulcerative colitis who developed progressive respiratory and constitutional symptoms while receiving infliximab. He developed severe hypoxaemic respiratory failure requiring high-flow nasal oxygen. Chest imaging demonstrated diffuse miliary nodularity with bilateral ground-glass changes, consolidation, and mediastinal lymphadenopathy. Despite previous negative and subsequent indeterminate QuantiFERON-TB results, the clinical and radiological findings prompted empirical anti-tuberculous treatment while respiratory samples were obtained. Sputum culture subsequently grew *Mycobacterium tuberculosis*, while a Hain molecular assay and bronchial washing confirmed the *M. tuberculosis* complex. Infliximab was withheld, and the patient improved clinically, with respiratory support reduced from high-flow nasal oxygen to conventional oxygen. He subsequently reported symptomatic improvement during respiratory follow-up. This case highlights the limitations of TB screening tests in patients receiving anti-TNF therapy and demonstrates that a negative or indeterminate interferon-gamma release assay (IGRA) should not delay investigation for active TB when clinical and radiological features are strongly suggestive.

## Introduction

Tumour necrosis factor-alpha (TNF-α) is central to host defence against *Mycobacterium tuberculosis*, including macrophage activation and maintenance of granulomatous inflammation. Inhibition of TNF-α therefore increases susceptibility to tuberculosis (TB) and can result in severe or disseminated disease. Infliximab has been particularly associated with TB [1].

Patients with inflammatory bowel disease should be assessed for TB infection before commencing anti-tumour necrosis factor (TNF) therapy, preferably before significant immunosuppression is established. This assessment should take into account individual risk factors and the epidemiological prevalence of TB, as anti-TNF treatment can increase the risk of reactivation or progression to active disease. Screening results should also be interpreted with caution in immunocompromised patients because immunosuppression may lead to false-negative tuberculin skin test results. The risk of TB in patients receiving infliximab or adalimumab also appears to vary according to the underlying TB burden of the population in which they live [2,3].

## Case presentation

A 32-year-old man with ulcerative colitis was receiving infliximab and mesalazine. His ulcerative colitis was clinically controlled, without a recent significant flare or rectal bleeding. Infliximab had been commenced at 10 mg/kg, with a second dose administered several weeks later.

He presented with approximately seven days of progressive shortness of breath and dry cough, accompanied by night sweats, intermittent feverish symptoms and about 3 kg of unintentional weight loss over the preceding three months. He denied known TB contact. He had previously travelled to Turkey, worked in a care-home setting and reported smoking/vaping.

At presentation, he was tachycardic; heart rate was 115 beats/min, blood pressure was 103/59 mm Hg, respiratory rate was 20/min, and oxygen saturation was 90% on room air. Although hypoxaemic, he did not initially appear markedly distressed. Cardiovascular examination was unremarkable, and the initial chest examination was relatively benign, although bilateral crepitations were subsequently noted. There was no peripheral lymphadenopathy, clubbing, or rash.

He was initially managed for severe chest infection with oxygen, intravenous fluids, and antibiotics. His oxygen requirement increased from conventional oxygen to 15 L/min via a non-rebreather mask and subsequently to high-flow nasal oxygen at up to 60 L/min with an inspired oxygen concentration of 70%. Despite this, he remained relatively comfortable and haemodynamically stable.

A chest radiograph showed widespread bilateral multiple small lung opacities consistent with miliary-type pulmonary infiltrates (Figure 1). This finding, together with his constitutional symptoms and recent initiation of infliximab therapy, prompted investigation for TB. These changes were not in his previous chest X-ray prior to infliximab therapy, which did not show any significant pulmonary abnormalities (Figure 2).

**Figure 1:**
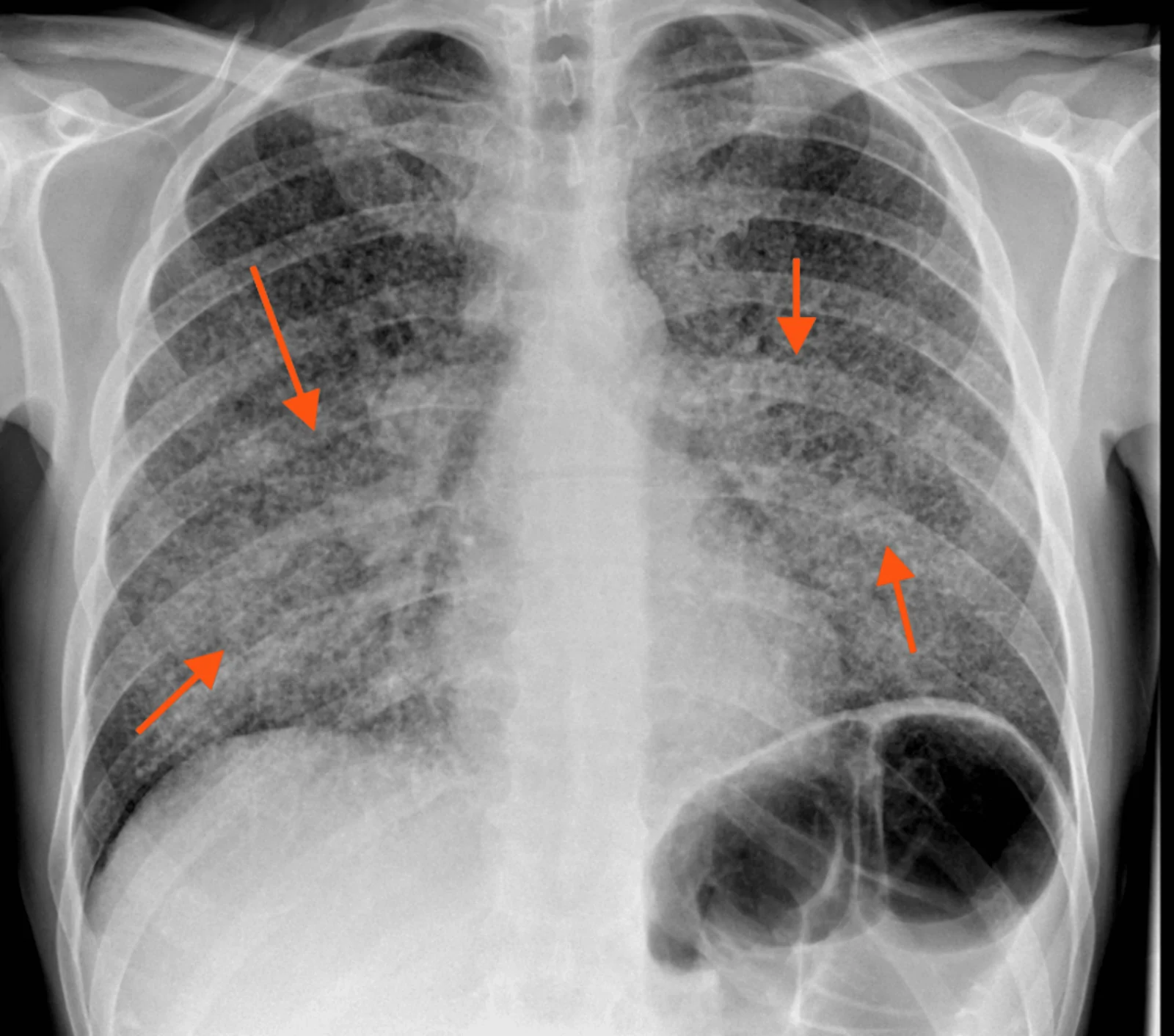
Chest radiograph showing diffuse bilateral miliary-pattern pulmonary nodularity (orange arrows).

**Figure 2:**
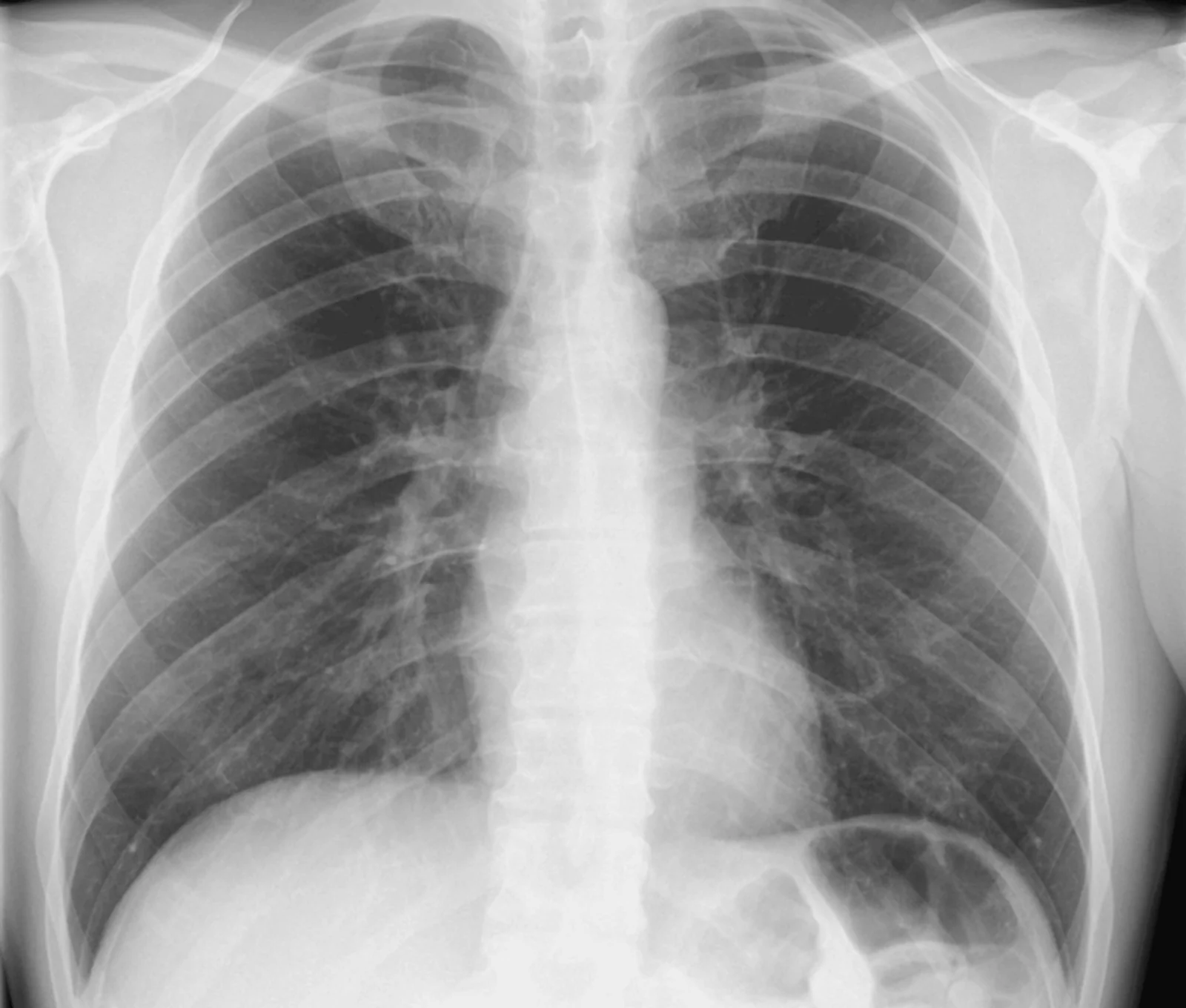
Chest radiograph obtained before initiation of infliximab showing no significant pulmonary abnormality.

Initial blood tests showed C-reactive protein (CRP) of 203 mg/L (reference range 0-8 mg/L), haemoglobin of 121 g/L (130-180 g/L), white cell count of 5.1 × 10⁹/L (4.0-10.0 × 10⁹/L), neutrophil count of 4.5 × 10⁹/L (2.0-7.0 × 10⁹/L), platelet count of 200 × 10⁹/L (150-410 × 10⁹/L), and sodium count of 131 mmol/L (133-146 mmol/L). Creatinine was 57 µmol/L (54-110 µmol/L) with preserved renal function, and D-dimer was 5,003 ng/mL (0-500 ng/mL). Arterial blood gas analysis demonstrated hypoxaemia without significant hypercapnia or lactic acidosis.

Respiratory viral testing for influenza A and B, respiratory syncytial virus and SARS-CoV-2 was negative. Legionella urinary antigen was negative, and blood cultures showed no growth. HIV, hepatitis B, and hepatitis C screening was negative.

CT pulmonary angiography excluded pulmonary embolism but demonstrated diffuse bilateral miliary nodularity with extensive ground-glass change and consolidation, together with enlarged mediastinal, subcarinal and right hilar lymph nodes. There was no pleural effusion (Figure 3).

**Figure 3:**
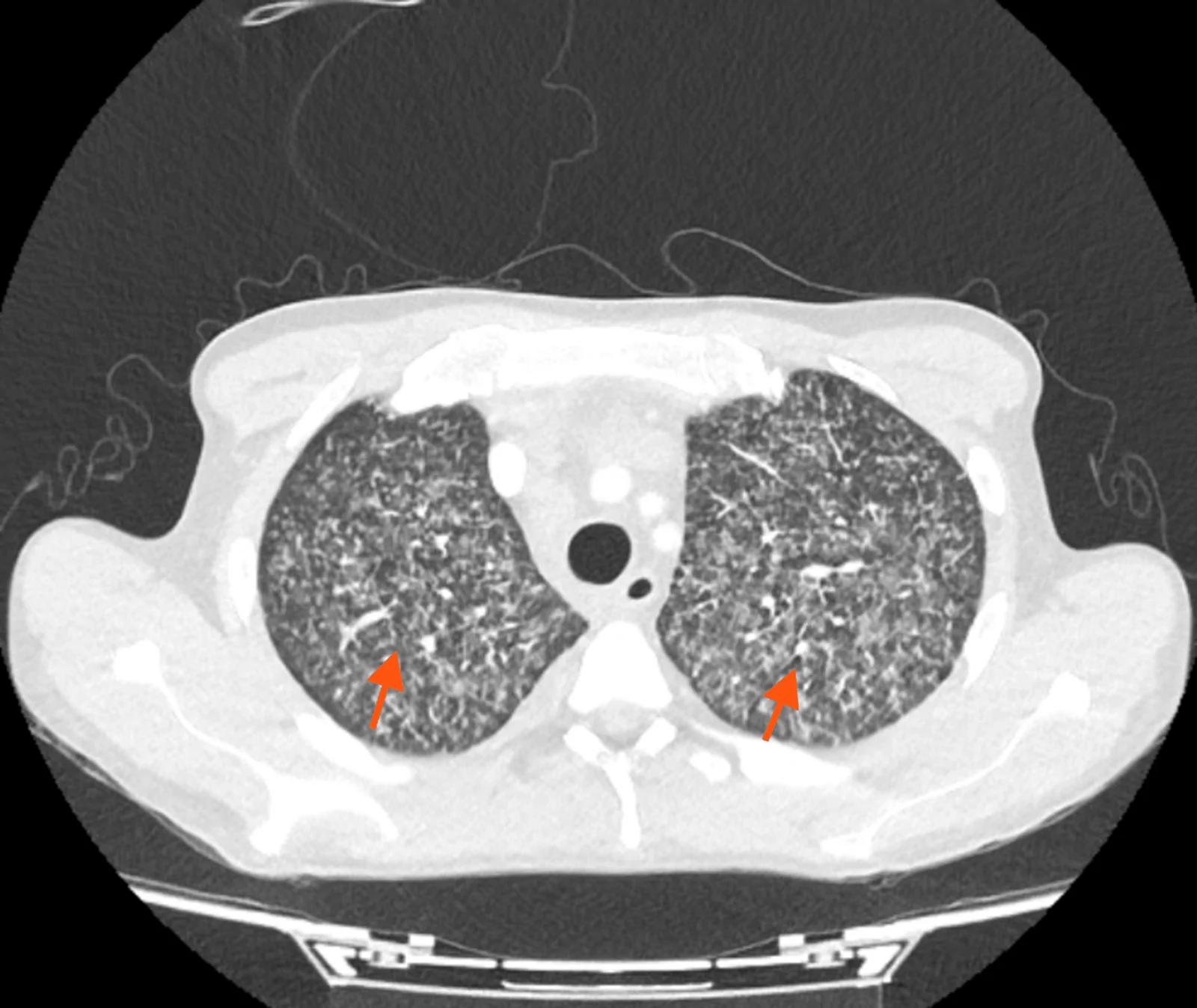
CT pulmonary angiogram showing diffuse bilateral miliary-pattern pulmonary nodularity with associated ground-glass opacities and areas of consolidation (orange arrows).

An autoimmune screen, including ANA, ANCA, and rheumatoid factor, together with an extended connective-tissue-disease antibody panel, was negative. *Aspergillus*-specific IgG was 3 mg/L (0-40 mg/L), *Aspergillus*-specific IgE was <0.1 (0-0.35), and total IgE was 62.7 kU/L (normal value less than 100 kU/L).

A pre-treatment QuantiFERON-TB test had been negative. Subsequent QuantiFERON-TB testing during the patient’s clinical deterioration was indeterminate, with a further inpatient test also returning an indeterminate result. In view of the characteristic radiological appearance and constitutional symptoms, a respiratory consultant advised empirical treatment for TB while respiratory samples were obtained.

Sputum mycobacterial culture subsequently identified *M. tuberculosis*. Acid-fast bacilli were not seen on microscopy; however, Hain molecular testing was positive. Bronchial washing from the left lung subsequently confirmed *M. tuberculosis* complex. Cytology showed mixed acute and chronic inflammatory cells, benign bronchial epithelial cells and occasional alveolar macrophages, with no malignant cells or granulomatous inflammation.

Differential diagnosis

The initial differential diagnosis included atypical pneumonia, infliximab-associated pneumonitis, vaping-associated lung injury, *Pneumocystis jirovecii* pneumonia, and other inflammatory or infective lung diseases. Atypical pneumonia was initially plausible because of the acute respiratory presentation and markedly raised CRP; therefore, broad-spectrum antibiotics were commenced. Infliximab-associated pneumonitis and vaping-associated lung injury were considered because of the bilateral ground-glass abnormalities and vaping history. However, neither diagnosis adequately explained the miliary pattern, constitutional symptoms and subsequent microbiological identification of *M. tuberculosis*. Pulmonary embolism was also considered because of the severe hypoxaemia and markedly elevated D-dimer but was excluded by CT pulmonary angiography.

Diagnosis

The diagnosis of microbiologically confirmed pulmonary TB with a miliary radiological pattern was established by the combination of compatible clinical and radiological findings and microbiological confirmation of *M. tuberculosis* in sputum and *M. tuberculosis* complex in bronchial washing. The negative and indeterminate QuantiFERON results did not exclude active TB. IGRAs assess immune sensitisation to TB antigens rather than directly detecting active infection, and their performance may be affected by immunosuppression and severe illness.

Management

Because of the high clinical and radiological suspicion of TB, empirical anti-tuberculous treatment was commenced before all microbiological results were available. The patient received Voractiv (a fixed-dose combination antibiotic tablet that contains rifampicin 150 mg, isoniazid 75 mg, pyrazinamide 400 mg, and ethambutol 275mg) with pyridoxine 10 mg once daily. Infliximab was withheld after active TB was suspected and subsequently confirmed. He was managed with appropriate respiratory isolation and multidisciplinary input.

Respiratory support was progressively reduced as his condition improved, with high-flow nasal oxygen being weaned to conventional nasal oxygen. After completion of the intensive phase, treatment was continued with rifampicin/isoniazid (Rifinah 300/150 mg, two tablets once daily) with pyridoxine 10 mg once daily.

The patient showed marked clinical and radiological improvement following initiation of anti-tuberculous therapy. His follow-up chest radiography demonstrated substantial resolution of the previously observed miliary-pattern pulmonary abnormalities (Figure 4).

**Figure 4:**
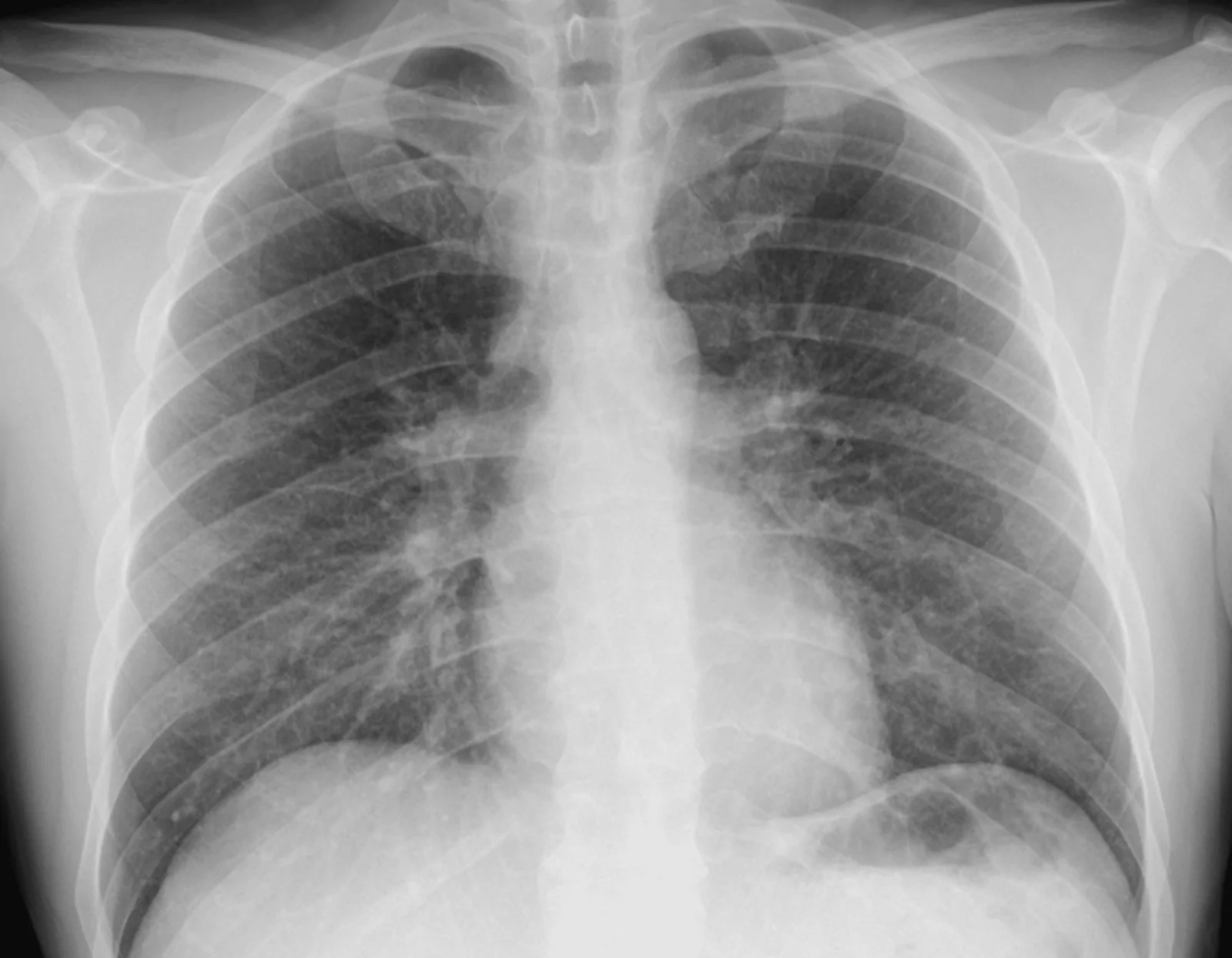
Follow-up chest radiograph showing substantial resolution of the previously demonstrated miliary-pattern pulmonary nodularity following anti-tuberculous therapy.

Outcome and follow-up

Following initiation of anti-tuberculous treatment and supportive care, the patient's respiratory status improved. His oxygen requirement decreased from high-flow support to conventional nasal oxygen, and he was clinically stable without significant respiratory distress at the documented stage of recovery.

The patient was subsequently reviewed after discharge in the respiratory clinic, where he reported an improvement in his respiratory symptoms and no unwanted side effects from anti-tuberculous treatment. He remains under respiratory specialist follow-up, with a further review planned with the respiratory consultant.

## Discussion

This case illustrates the diagnostic challenge of TB in a patient receiving TNF-α inhibition. TNF-α has an important role in host defence against *M. tuberculosis*, including macrophage activation and maintenance of granulomatous containment of mycobacteria. Consequently, pharmacological inhibition of TNF-α increases susceptibility to TB and may alter the clinical presentation of infection [1,2]. Early post-marketing experience with infliximab demonstrated that TB occurring during treatment could be severe and was frequently associated with extrapulmonary or disseminated disease [1].

The occurrence of TB despite apparently reassuring pre-treatment screening is an important feature of this case. A systematic review and meta-analysis of patients with inflammatory bowel disease receiving infliximab or adalimumab identified 373 cases of TB among 130,114 treated patients. Notably, 73% of patients who developed TB had no evidence of latent TB infection on pre-treatment screening [3]. These findings demonstrate that pre-treatment latent TB infection screening does not completely exclude the possibility of subsequent active TB during anti-TNF therapy [3].

TB developing despite negative pre-treatment screening has also been described in inflammatory bowel disease (IBD) cohorts. Jauregui-Amezaga et al. reported patients who developed TB during anti-TNF therapy despite negative assessment for latent TB infection (LTBI), including cases occurring relatively soon after treatment initiation [4]. Similarly, the GETAID cohort identified 44 patients with IBD who developed TB despite negative pre-treatment screening. Pulmonary involvement was reported in 57% of cases and extrapulmonary involvement in 91%, while potential epidemiological exposures included healthcare-related occupational exposure and previous travel to countries with higher TB prevalence [5]. Other reports have described disseminated or miliary TB despite negative screening before anti-TNF therapy [6,7]. The present case therefore adds to existing evidence that a negative baseline screening assessment cannot be regarded as excluding subsequent active TB during anti-TNF treatment.

The sequence of TB screening results in this patient is particularly relevant. Interferon-gamma release assays, including QuantiFERON-TB Gold, assess an immune response to *M. tuberculosis*-associated antigens rather than detecting viable organisms directly. Their performance may therefore be affected when cellular immune function is altered by disease or immunosuppressive treatment [8]. A systematic review and meta-analysis in patients with IBD found that immunosuppressive therapy was associated with a lower likelihood of a positive IGRA result and a higher frequency of indeterminate results [9]. In particular, the pooled analysis demonstrated an increased risk of indeterminate IGRA results among patients receiving immunosuppressive therapy, highlighting a limitation of relying on immunological testing alone in immunosuppressed patients [9].

The present case is also notable because a previous US IBD cohort described a patient who had an indeterminate QuantiFERON result while receiving infliximab and subsequently developed miliary TB [10]. An indeterminate IGRA should therefore be interpreted as an unresolved result rather than as evidence that TB infection is absent, particularly when there are compatible clinical or radiological findings.

The circumstances in which the later QuantiFERON test was performed may also have reduced its diagnostic usefulness. In a study of hospitalised patients with IBD, indeterminate QuantiFERON results were substantially more frequent among inpatients than outpatients, with 52.7% of inpatients compared with 3.14% of outpatients having an indeterminate result [11]. Inpatient status and steroid exposure were independently associated with indeterminate results, while inflammatory laboratory abnormalities were also more frequently observed in patients with indeterminate tests [11]. These findings are relevant to the interpretation of an indeterminate result obtained during acute severe illness. In a patient with constitutional symptoms, severe respiratory compromise and radiological features suggestive of TB, an indeterminate IGRA should not provide reassurance against active infection.

Imaging played an important role in raising suspicion for pulmonary TB in this case. The chest CT demonstrated widespread small pulmonary nodules with associated ground-glass change and areas of consolidation, together with mediastinal and hilar lymphadenopathy. A diffuse micronodular or miliary pattern is recognised as a possible manifestation of TB, although the radiological appearance is not specific. The differential diagnosis in this patient appropriately included drug-related pneumonitis, vaping-associated lung injury, *Pneumocystis jirovecii* pneumonia, and other atypical infections. The subsequent microbiological identification of *M. tuberculosis* in respiratory specimens was therefore essential in distinguishing active TB from these alternative diagnoses.

The absence of granulomatous inflammation on bronchial cytology did not exclude TB in this patient, as the diagnosis was established microbiologically from respiratory specimens. Histological or cytological appearances may be altered in patients receiving immunosuppressive therapy and the absence of a classical granulomatous response does not exclude mycobacterial infection. In this patient, microbiological investigation was decisive: *M. tuberculosis* was identified from sputum, with subsequent confirmation of the *M. tuberculosis* complex in bronchial washing. The microbiological findings therefore established active pulmonary TB despite the absence of granulomatous inflammation on cytological examination. 

The negative pre-treatment screening result does not allow the mechanism of subsequent TB to be determined. The infection may have represented reactivation of previously unrecognised infection or acquisition after baseline screening.

The timing of the illness in relation to infliximab exposure is also clinically important. TB associated with anti-TNF therapy has been reported relatively early after treatment initiation and miliary TB has been described in patients receiving infliximab despite apparently negative pre-treatment screening [1,4,7,12]. TNF-α inhibition provides a biologically plausible mechanism through which previously contained *M. tuberculosis* infection could progress.

The patient's epidemiological history further demonstrates why risk assessment should continue after initiation of biologic therapy. Previous travel to Turkey and employment in a care-home environment were relevant considerations when assessing possible exposure to TB. In the GETAID cohort, occupational exposure and previous travel were identified among potential epidemiological factors in patients who subsequently developed TB despite negative baseline screening [5]. In addition, prospective studies of patients receiving anti-TNF therapy have demonstrated changes or conversion in TB immunological test results during follow-up, supporting the importance of reassessing epidemiological and clinical risk rather than relying solely on a historical screening result [13]. The 2026 European Crohn's and Colitis Organisation (ECCO) guidance similarly recommends TB screening before anti-TNF therapy and recognises the potential need for repeat assessment in patients with ongoing or new risk factors, including substantial exposure to respiratory TB and patients whose baseline testing was undertaken while immunosuppressed [14].

Empirical anti-tuberculous treatment was considered justified in this case because the probability of clinically significant TB was high before microbiological confirmation was available. The combination of severe hypoxaemic respiratory failure, constitutional symptoms, recent weight loss, a characteristic diffuse pulmonary radiological pattern and current anti-TNF exposure created a high-risk clinical picture. Waiting for definitive culture confirmation in a patient with suspected severe or potentially disseminated TB could have resulted in further clinical deterioration. Previous reports of miliary and severe TB during infliximab therapy support maintaining a low threshold for microbiological investigation and treatment when clinical suspicion is high [7,12]. Current ECCO guidance also emphasises assessment for TB and other infections before and during advanced IBD therapy, particularly in patients receiving anti-TNF agents [14].

Infliximab was withheld once active TB was suspected and subsequently confirmed. This is consistent with the principle that TNF-α inhibition should not be continued during serious active infection and with current guidance concerning management of infections in patients receiving advanced IBD therapies [14]. Decisions regarding subsequent reintroduction of biologic therapy should be individualised according to microbiological and clinical control of TB, the activity and severity of the underlying IBD, alternative therapeutic options and the patient's overall risk profile, with appropriate gastroenterology and infectious diseases or respiratory specialist input [14].

This case has several limitations. The sequence of negative and indeterminate IGRA results cannot establish whether the patient had latent TB before infliximab initiation or acquired TB subsequently. In addition, the diagnosis was based on respiratory microbiological confirmation and characteristic radiological findings; extrapulmonary disease was not demonstrated in the information available. Therefore, the case should not be interpreted as proving failure of pre-treatment screening to detect latent infection in this individual, but rather as illustrating that negative or indeterminate immunological testing cannot exclude active TB when clinical suspicion is high.

The early clinical outcome in this case was favourable from a respiratory perspective. The patient improved sufficiently to progress from high-flow nasal oxygen to conventional oxygen therapy and subsequently reported symptomatic improvement during respiratory clinic follow-up, without unwanted effects from anti-tuberculous treatment.

## Conclusions

Miliary pulmonary TB should remain an important differential diagnosis in patients receiving infliximab who develop constitutional symptoms, hypoxaemic respiratory failure, and diffuse pulmonary abnormalities. Pre-treatment screening for latent TB is an important risk-reduction strategy, but a negative or indeterminate QuantiFERON result does not exclude active TB, particularly in patients receiving immunosuppressive therapy or those who are acutely unwell.

Clinical assessment, epidemiological risk factors, radiological findings, and microbiological investigation should therefore be considered together. In patients with high clinical and radiological suspicion of active TB, a negative or indeterminate screening test should not delay appropriate respiratory sampling and, when clinically justified, empirical treatment while definitive microbiological results are awaited.

## References

[1] Keane J, Gershon S, Wise RP (2001). Tuberculosis associated with infliximab, a tumor necrosis factor alpha-neutralizing agent. N Engl J Med.

[2] Gardam MA, Keystone EC, Menzies R (2003). Anti-tumour necrosis factor agents and tuberculosis risk: mechanisms of action and clinical management. Lancet Infect Dis.

[3] Kedia S, Mouli VP, Kamat N, Sankar J, Ananthakrishnan A, Makharia G, Ahuja V (2020). Risk of tuberculosis in patients with inflammatory bowel disease on infliximab or adalimumab is dependent on the local disease burden of tuberculosis: a systematic review and meta-analysis. Am J Gastroenterol.

[4] Jauregui-Amezaga A, Turon F, Ordás I, Gallego M, Feu F, Ricart E, Panés J (2013). Risk of developing tuberculosis under anti-TNF treatment despite latent infection screening. J Crohns Colitis.

[5] Abitbol Y, Laharie D, Cosnes J (2016). Negative screening does not rule out the risk of tuberculosis in patients with inflammatory bowel disease undergoing anti-TNF treatment: a descriptive study on the GETAID cohort. J Crohns Colitis.

[6] Debeuckelaere C, De Munter P, Van Bleyenbergh P, De Wever W, Van Assche G, Rutgeerts P, Vermeire S (2014). Tuberculosis infection following anti-TNF therapy in inflammatory bowel disease, despite negative screening. J Crohns Colitis.

[7] Carvalho LP, Túlio MA, Rodrigues JP, Bana E Costa T, Chagas C (2018). Miliary tuberculosis in a Crohn's disease patient: the risk beyond the screening. GE Port J Gastroenterol.

[8] Pai M, Behr M (2016). Latent Mycobacterium tuberculosis infection and interferon-gamma release assays. Microbiol Spectr.

[9] Park CH, Park JH, Jung YS (2022). Impact of immunosuppressive therapy on the performance of latent tuberculosis screening tests in patients with inflammatory bowel disease: a systematic review and meta-analysis. J Pers Med.

[10] Qumseya BJ, Ananthakrishnan AN, Skaros S (2011). QuantiFERON TB gold testing for tuberculosis screening in an inflammatory bowel disease cohort in the United States. Inflamm Bowel Dis.

[11] Lovatt J, Gascoyne-Binzi D, Hussey T, Garside M, McGill F, Selinger CP (2021). Screening for TB in hospitalised patients with inflammatory bowel disease before anti-TNF therapy: is QuantiFERON Gold testing useful?. J Clin Med.

[12] Stas P, D'Hoore A, Van Assche G (2006). Miliary tuberculosis following infliximab therapy for Crohn disease: a case report and review of the literature. Acta Gastroenterol Belg.

[13] Abreu C, Afonso J, Camila Dias C, Ruas R, Sarmento A, Magro F (2017). Serial tuberculosis screening in inflammatory bowel disease patients receiving anti-TNFα therapy. J Crohns Colitis.

[14] Yanai H, Ellul P, Verstockt B (2026). ECCO guidelines on the prevention, diagnosis, and management of infections in inflammatory bowel disease. J Crohns Colitis.

